# Temporal Changes in Clinical Outcomes after Minimally Invasive Surgery for Hallux Valgus Correction in Women without Postoperative Complications

**DOI:** 10.3390/jcm12134368

**Published:** 2023-06-28

**Authors:** Luci M. Motta, Ignacio Manchado, Gustavo Blanco, María P. Quintana-Montesdeoca, Laura Garcés, Gerardo L. Garcés

**Affiliations:** 1 Hospital Perpetuo Socorro, 35007 Las Palmas, Spain; luci.motta@traumaquir.es (L.M.M.); nachomanchado@gmail.com (I.M.); gustblan@gmail.com (G.B.); 2 Department of Ciencias Médicas y Quirúrgicas, University of Las Palmas de Gran Canaria, 35007 Las Palmas, Spain; 3 Department of Mathematics, University of Las Palmas de Gran Canaria, 35007 Las Palmas, Spain; mariadelpino.quintana@ulpgc.es; 4 Terapias Acuáticas Canarias SL, 35007 Las Palmas, Spain; garcslaura@gmail.com

**Keywords:** AOFAS score, clinical outcomes, hallux valgus, MIS correction, MOXFQ

## Abstract

Minimally invasive surgery (MIS) is currently used to correct hallux valgus deformities. Most studies reporting on MIS techniques to correct hallux valgus deformities included patients with postoperative complications. These reported complications, with an average rate of 23%, had significant negative effects on the clinical outcomes in this patient population. In the present study, a cohort of 63 women who underwent MIS hallux valgus correction was assessed preoperatively and at a mean follow-up of 1.0, 4.7, and 6.5 years using the American Orthopaedic Foot and Ankle Society (AOFAS) scale and the Manchester Oxford Foot Questionnaire (MOXFQ). The main criterion for inclusion in this cohort was a lack of complications during the entire follow-up period. The results showed significant improvements in both AOFAS and MOXFQ scores between the preoperative and 1-year follow-up assessments. By contrast, clinically small and nonsignificant changes were observed among postoperative follow-up values. The number of enrolled patients needs to be increased in future studies, with different surgeons and techniques included. Nevertheless, our study findings will inform patients about the outcomes they can expect over the years if no complications occur.

## 1. Introduction

Hallux valgus (HV), a common condition that results from a complex positional deformity of the first ray, can lead to considerable pain and altered joint mechanics. Predisposing factors include female sex, age, inadequate footwear, and family history of HV. Women younger than 65 years with HV have a significantly worse quality of life than age-matched women in the general population [1,2]. Nonsurgical treatments include shoe modifications, pads, orthotic devices, and activity modifications. Surgery is considered when nonsurgical treatments fail or for cosmetic reasons. The goals of surgical treatment are pain relief, correction of the deformity, improved first-ray stability, and improved quality of life [3].

More than 100 surgical procedures are available for correcting HV deformities. Most procedures are performed using open techniques, but in the last two decades, percutaneous and minimally invasive surgery (MIS) approaches tended to be used. Their theoretical advantages include smaller scars, less postoperative pain, rapid recovery, reduced rehabilitation time, and decreased risk of infection [4,5,6,7,8]. Concerns regarding MIS use include a difficult learning curve, imprecise results, the necessity of fluoroscopy, higher economic costs, and an increased frequency of cutaneous complications [6,8,9]. Several changes have been implemented from the first-generation to the current third-generation MIS techniques. Inshall [10] initially described a percutaneous osteotomy without internal fixation. Due to the difficulties in keeping the reduction after the correction, there is a tendency to fix the osteotomy with percutaneous screws [11,12] or with a more sophisticated minimally invasive intramedullary nail device (MIIND) [13]. Despite initial concerns regarding the reproducibility of surgical outcomes, current MIS and open techniques offer similar clinical and radiological results [7,14,15,16,17,18,19].

In the past, authors have reported HV surgery results considering the radiological correction of deformities. However, sufficient evidence now suggests that radiological improvement is unrelated to patient satisfaction or clinical outcomes [1,16,20,21]. Distal linear metatarsal osteotomy improves foot-related quality of life in patients with HV deformity despite a high rate of postoperative radiographic complications, especially hallux varus [22]. Even when surgery is performed by an experienced surgeon, the potentials for patient dissatisfaction and unfavourable outcomes remain despite adequate radiological correction of the deformities [23].

Functional and subjective assessments are essential for measuring clinical results. Several questionnaires and scales have been used to measure functional clinical outcomes after HV surgery. The American Orthopaedic Foot and Ankle Society (AOFAS) scale, ranging from 0 to 100, is most frequently used [16,24,25]. Its disadvantages include the incorporation of physical examination parameters that have shown poor inter- and intra-observer reliability, the lack of self-administration thereby increasing the risk of bias, and the use of a single question regarding pain [25]. However, several authors reported significant improvements in AOFAS scores after MIS for HV correction [4,9,20,26,27,28].

Patient-reported outcome measures (PROMs) are preferred because they directly provide information, are independent of the surgeon’s perspective, and aid in minimizing bias of the surgical team [25]. Several PROM questionnaires are available, and the Manchester Oxford Foot Questionnaire (MOXFQ) is the most commonly used [29,30]. It consists of 16 questions with a score of 100 for three separate domains, although the three domain scores can be summarized as a single index score [31]. The MOXFQ properties have been validated in different studies concerning HV pathology and have shown good reliability, validity, and responsiveness [25,32].

Studies examining MOXFQ scores after MIS for HV correction demonstrated that all patients reported significant improvements between preoperative and final follow-up states. Most authors followed their patients for 2 years or less [18,33,34,35,36], but some followed them for at least 5 years [20,37]. All these studies compare the preoperative values with those of the final follow-up. However, it is unclear whether the results would differ if they were analysed at different time points. Lewis et al. [38] studied changes over time in MOXFQ scores of 202 feet operated using MIS techniques and found that the majority of improvements occurred within the first 6 months. A subgroup of 17 feet (8.4%) had worse MOXFQ index scores 6 months following minimally invasive chevron and Akin osteotomy (MICA); of these, 14 feet (82.4%) showed a significant improvement in the MOXFQ index score 2 years after the operation compared to their preoperative score.

Worldwide, healthcare delivery has been increasingly prioritizing patient satisfaction. Up to 10% of patients are dissatisfied with the results of HV surgery [39]. Therefore, it is necessary to broaden the search for predictors of patient-reported outcomes after HV correction. Several psychological, clinical, and social factors influence these outcomes. Complications such as infections, nonunion of osteotomies, hardware-related problems, and thrombophlebitis have considerable negative effects on correction outcomes [36,40]. These complications are infrequent but should be explained to patients. Most patients are informed of the expected outcomes if no major complications occur. However, little is known about the outcomes in cohorts of patients without complications.

Understanding how functional and PROM scores change over time following MIS HV surgery and whether this change is clinically meaningful will help surgeons counsel patients, reassure surgeons, and predict the likelihood of improvement at a certain time point [38]. Patients should be advised about possible surgery complications; however, informing them about the expected results if no complication occurs could be of great utility. The aim of this study was to investigate changes over time in functional (AOFAS) and PROM (MOXFQ) scores in a cohort of women who underwent MIS to correct HV deformities and had no operation-related complications during follow-up. Our hypothesis was that no significant changes should be expected in clinical outcomes later than 1 year after the operation if no complications occurred.

## 2. Materials and Methods

### 2.1. Study Design

This retrospective study comprised 73 female patients who underwent MIS to correct unilateral HV deformities performed by the same surgical team between 2013 and 2014. Laterality and morphological type of the operated foot were considered as variables. The median patient age was 57 (interquartile range [IQR]: 46–63; range: 22–77) years.

Patients were initially included in the study if they were women older than 18 years with a unilateral radiological HV angle > 20° that caused discomfort or pain during activities of daily living. Exclusion criteria were bilateral symptomatic HV, a previous operation or fracture on the affected foot, hallux rigidus, general or local inflammatory, neurological, or vascular disease, and lesser toe deformities on the same foot subsidiaries of surgical correction. Patients with asymptomatic contralateral HV were also excluded.

### 2.2. Type of Operation

The operations were performed using a modified MIS technique based on the Isham–Reverdin procedure [10]. This technique was described in detail by Biz et al. [4]. In summary, a peri-malleolar tibialis and peroneal nerves block was made under local anaesthesia (15–20 cc of 50% bupivacaine-mepivacaine solution) and sedation, and three percutaneous incisions of 3–4 mm were made under fluoroscopic vision. The first was at the level of the first metatarsal neck to allow bunionectomy and transverse osteotomy of the metatarsal bone just proximal to the sesamoid level. Contrary to the technique of Biz et al. [4], we pushed out the metatarsal head to translate it laterally, approximately one-fourth of the osteotomy line length, thus closing the intermetatarsal space. The sesamoid position was not checked after the metatarsal head displacement. Through a second dorsal incision, the lateral soft tissue and transverse head of the abductor hallucis were released immediately, lateral to the metatarsophalangeal joint. In some cases, through a third incision, an incomplete medial transverse osteotomy of the proximal phalange (Akin osteotomy) was performed 1 cm distal to the articular line to improve the HV angle. Internal fixation was not performed. The correction was retained with a dressing around the hallux under fluoroscopic vision. Immediate weight bearing was allowed postoperatively using a shoe with a flat, stiff sole, and the dressing was changed every 2 weeks for 8 weeks after the operation.

### 2.3. Assessment Method

To assess functional outcome and pain the AOFAS score was used. To assess patient reported outcome after corrective surgery the MOXFQ was used. Using the AOFAS Hallux Metatarsophalangeal–Interphalangeal scale [41,42] and the MOXFQ [43], with specific values of the three subscales of this questionnaire—Walking–Standing (W-S), Pain, and Social Interaction (S-I)—patient scores were obtained at 4–5 days preoperatively and at a mean of 1.0, 4.7, and 6.5 years postoperatively (Table 1). Values were differentiated regarding the laterality and the morphological type of the affected foot. Standard weight-bearing dorsoplantar and lateral views were acquired to verify the bone union at the osteotomy site 2 months after the operation or during follow-up in cases of persistent pain or recurrence of the deformity.

### 2.4. Statistical Analysis

Statistical analyses were performed using SPSS Statistics v27.0 for Windows (IBM Corp., Armonk, NY, USA). Categorical variables were summarized using absolute frequencies and percentages. The chi-square test was used to analyse the association between two categorical variables. The numerical variables were summarized using the mean, standard deviation, minimum, and maximum. The Shapiro–Wilk test was used to check for normality. To compare the preoperative status of the patients according to the laterality of the foot, Student’s *t*-test for two independent samples or the nonparametric Mann–Whitney U test was used, depending on whether the condition of normality of the data was met. Furthermore, to assess whether the preoperative baseline measurements were independent of the three-foot types, ANOVA or the nonparametric Kruskal–Wallis test was used, depending on whether the normality of the samples was confirmed. The non-parametric Wilcoxon test was used to compare the pre-operative and post-operative (at 6.5 years) scores according to the laterality and morphological type of the affected foot. General linear-model repeated measures were used to analyse whether the evolution of the patients according to the numerical data varied from the preoperative assessment to the third postoperative control, considering time as an intra-subject factor (four levels). Results were considered statistically significant if *p*-values were <0.05.

## 3. Results

From the initial 73 operated patients, 2 were excluded due to deep infection of the operative site, one due to thrombophlebitis of the operated limb, 1 due to death of the patient unrelated to HV surgery, 1 due to nonunion of the osteotomy, 2 who needed reoperation due to transfer metatarsalgia, 1 due to insufficient correction, and 2 who were lost to follow-up. The final patient population consisted of 63 women between 28 and 73 (mean age 50.3 ± 12.8) years, who presented no complications during the observation period. Table 2 summarizes the morphological characteristics of the affected feet. No significant association was found between foot type and laterality (*p* = 0.964).

The preoperative MOXFQ and AOFAS scores of the patients showed no significant differences regarding the laterality (Table 3) or the morphological type of the affected foot (Table 4).

Similarly, the preoperative W-S, Pain, and S-I scores showed no significant differences regarding the laterality (Table 5) or the morphological type of the affected foot (Table 6).

**Figure 1:**
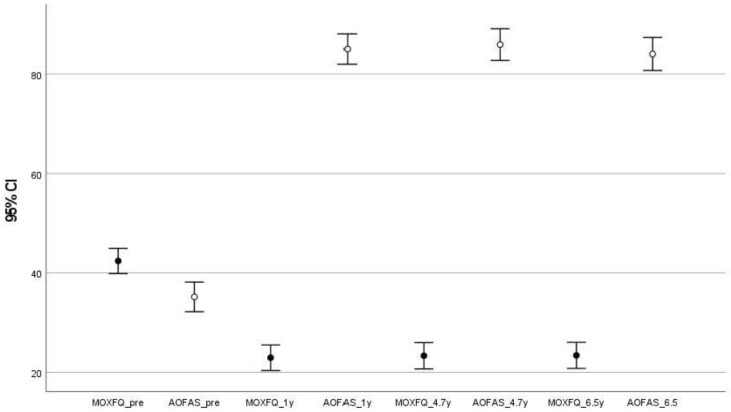
AOFAS and MOXFQ scores with their 95% CIs at pre- and postoperative time points. AOFAS, American Orthopaedic Foot and Ankle Society; CI, confidence interval; MOXFQ; Manchester Oxford Foot Questionnaire; y, years.

Table 7 summarizes and compares the pre- and postoperative AOFAS and MOXFQ scores. The AOFAS score increased by nearly 50 points from the preoperative level at all postoperative time points (*p* < 0.001); F = 223.1; df = 3; eta partial squared= 0.918; observed power = 1 (Multivariate test). The three postoperative scores differed by less than 2 points, although the values were significantly different between postoperative years 4.7 and 6.5 (*p* = 0.002). The MOXFQ score decreased by nearly 20 points from the preoperative level at all postoperative time points (*p* < 0.001); F = 61.8; df = 3; eta partial squared = 0.756; observed power = 1 (Multivariate test). Differences in MOXFQ scores among the three postoperative values did not exceed 1 point, although the values were again significantly different between postoperative years 4.7 and 6.5 (*p* = 0.048). The 95% confidence intervals (CIs) are shown in Figure 1. Time had a significant effect on the scores of both scales (*p* < 0.001). Mauchly’s sphericity test indicated that the assumption of sphericity was violated (*p* < 0.001), and the Greenhouse–Geisser correction was used in both cases. The eta partial squared value was 0.732 (Observed power = 1) for the MOXFQ scale and 0.893 (Observed power = 1) for the AOFAS scale, which implies a large effect size for each scale.

**Figure 2:**
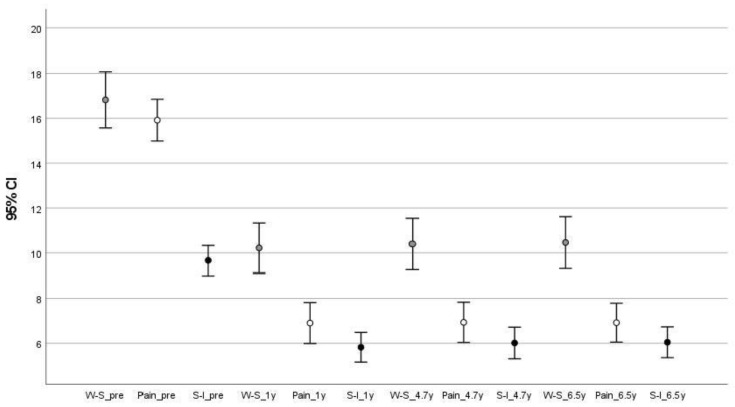
Scores of the three MOXFQ subscales with their 95% CIs at pre- and postoperative time points. CI, confidence interval; MOXFQ; Manchester Oxford Foot Questionnaire; S-I, Social Interaction; W-S, Walking–Standing; y, years.

Table 8 shows the scores of the three MOXFQ subscales at pre- and postoperative time points. According to the multivariate test there was a significant effect of time for Walking–Standing (F = 31.79; df = 3; eta partial squared = 0.614; observed power = 1), for Pain (F = 90.26; df = 3; eta partial squared = 0.819; observed power = 1) and for the Social Interact values (F = 34.54; df = 3; eta partial squared = 0.633; observed power = 1). The difference between the preoperative W-S score and all postoperative W-S scores was about 6 points (*p* < 0.001), whereas the postoperative W-S scores differed by 0.2 points or less (not significant). Likewise, the difference between the preoperative Pain score and all postoperative Pain scores was 6 points (*p* < 0.001); there were no differences among the postoperative pain scores. The differences between the preoperative S-I score and all postoperative S-I scores ranged between 3.6 and 3.8 (*p* < 0.001). The maximum difference among postoperative S-I scores was 0.2 points (not significant). The 95% CIs are shown in Figure 2. Mauchly’s sphericity test indicated that the assumption of sphericity was violated (*p* < 0.001), and the Greenhouse–Geisser correction was used for each of the three variables. The eta partial squared value was 0.593 (Observed power = 1) for the W-S item, 0.804 (Observed power = 1) for the Pain item, and 0.581 (Observed power = 1) for the S-I item. In all cases, a large effect size was confirmed.

Differences in pre- vs. post-operative (6.5 years) AOFAS and MOXFQ scores regarding the laterality of the affected foot (Table 9) and the morphological type of the affected foot (Table 10) were highly significant.

Differences in pre- vs. post-operative (6.5 years) subscales of the MOXFQ scores regarding the laterality of the affected foot (Table 11) and the morphological type of the affected foot (Table 12) were highly significant.

**Figure 3:**
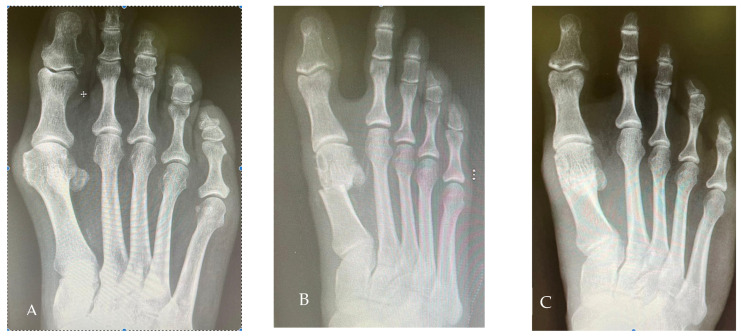
Radiological views of one case. (**A**): Preoperative; (**B**): Two weeks after the operation; (**C**): Two years after the operation.

**Figure 4:**
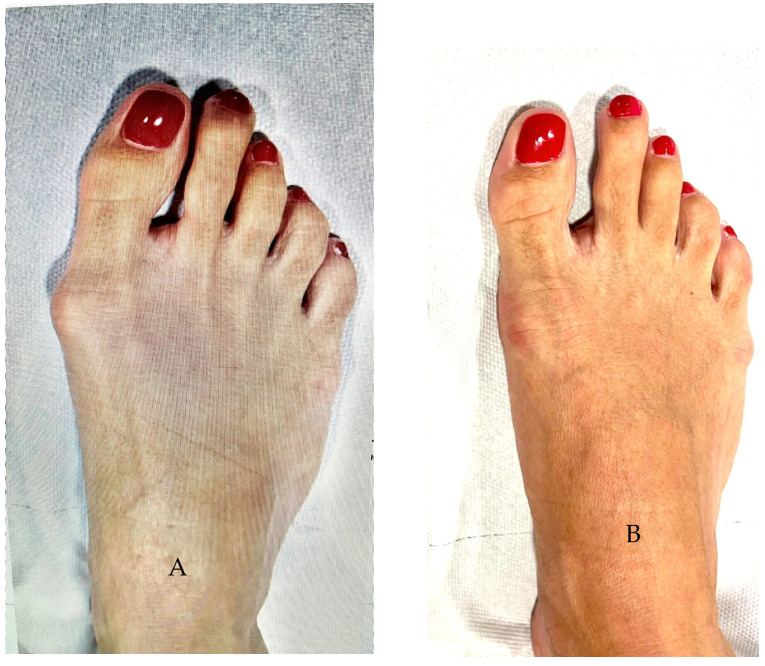
Clinical views of the same case of Figure 3. (**A**): Preoperative; (**B**): Two years after the operation.

Some examples of pre- to post-operative radiological and clinical changes are shown in Figure 3 and Figure 4.

## 4. Discussion

The main finding of this work is that both AOFAS and MOXFQ scores significantly improved 1 year after MIS correction of HV and that this correction was maintained without significant changes 4.7 and 6.5 years later. Moreover, the scores of the three MOXFQ subscales also significantly improved 1 year after the operation and remained at this level without significant changes during follow-ups until 6.5 years postoperatively.

Our results showed a significant difference in the AOFAS score from the preoperative to the final follow-up value at 6.5 years (35.1 vs. 84.0 points, respectively). This difference was similar to that observed by other authors after MIS correction. Biz et al. [4] reported a score of 87.15 points 48 months after surgery representing an increase of 33 points. With the use of a MIIND, Biz et al. [13] found an improvement in AOFAS score from 57.9 preoperatively to 90.5 after a mean of 96 months postoperatively. Motta et al. [20] found a pre-to-post difference of 54 points 6 years after the operation (from 35 to 90 points). Castellini et al. [9] reported pre- to postoperative changes in AOFAS scores from 47.3 to 87.0 points, respectively, 2 years after surgery. Xu et al. [19] found that the AOFAS score increased from preoperative 44.0 points to postoperative 90.2 points. Del Vecchio et al. [44] observed a pre-to-post change from 52.1 to 92.1 points. For a systematic review, Miranda et al. [40] selected 16 publications including 1246 patients with MIS for HV correction. The mean AOFAS scores improved from pre- to postoperative values ranging from 51.0 to 89.3 points. In a recent meta-analysis, Alimy et al. [14] selected seven studies (395 feet) including six randomized controlled studies and one prospective comparative study. They reported a final AOFAS score of 88 ± 7 points. Caravelli et al. ([8] reported four studies (464 cases) with a mean final AOFAS score of 90.2.

In our study, the MOXFQ scores improved from 42.4 points preoperatively to 23.4 points 6 years later. This difference was smaller than that reported by other studies on MIS HV correction. Del Vecchio et al. [44] observed a preoperative score of 40 points, which changed to 5.3 points at follow-up 18 months later. Patnaik et al. [18] found a pre-to-post change in MOXFQ score of 64.6 vs. 11.6 points 2 years later. Likewise, Lewis et al. [38] reported a score of 40.6 points in the preoperative control and 6.7 points 2 years later, and Lewis et al. [37] observed a MOXFQ index score of 2.3 points 5 years after the operation.

Our results showed a differential development from preoperative to final follow-up scores among the three MOXFQ domains, i.e., 16.8 vs. 10.4 points for W-S, 15.9 vs. 6.9 points for Pain, and 9.6 vs. 6.0 points for S-I. These results differ from those of Lewis et al. [38], who observed at a minimum 2-year follow-up that the MOXFQ scores had significantly improved in each domain, i.e., they decreased from 44.5 points preoperatively to 9.4 points postoperatively for Pain, from 38.7 to 6.5 points for W-S, and from 48.0 to 6.6 points for S-I. In addition, Lewis et al. [36] reported that 2 years after surgery, the MOXFQ scores significantly improved in the Pain, W-S, and S-I domains from 39.2 to 7.5 points, 38.2 to 5.9 points, and 48.6 to 5.5 points, respectively.

Few authors have reported results at multiple time points in the same cohort of patients after MIS correction of HV [4,45,46]. Apart from Lewis et al. [38], little attention has been paid to changes over time. The majority of PROM improvements following MIS correction are achieved by 6 months postoperatively, but a further small significant improvement can be seen up to 2 years [36]. Other authors have reported similar findings but without reaching statistical significance [47]. Biz et al. [4] observed at different follow-up time points that the mean total AOFAS score of MIS-treated patients improved progressively and significantly: 54.1 points before surgery, 72.2 points at 3-month follow-up, 78.6 points at 12-month follow-up, and 87.2 points at the final 48 months-follow-up. Using a MIINF, Biz et al. [13] observed a progression of the AOFAS score from 26.2 at pre-operation to 69.6 at 6 months, 81.4 at 12 months, and 87.6 at a mean of 96 months post-operatively.

We found only one report that studied the changes in MOXFQ scores over time in a cohort of patients after MIS correction for HV [38]. These authors evaluated 202 feet with complete PROM data. They found a statistically significant improvement in the MOXFQ index score at each time point following MICA surgery for up to 2 years of follow-up. However, the differences after 6 months may not be clinically significant. Our study also showed significant differences in MOXFQ scores between pre- and postoperative time points, but the differences among the three postoperative values were less than 1 point and not significant. Our patients also showed significant differences in AOFAS scores between the preoperative and follow-up time points. Although a significant difference of nearly 2 points in the AOFAS score between the 4.7-year and 6.5-year follow-ups was found, this difference is probably not clinically relevant. Chan et al. [48] observed 2 years after HV correction that the mean AOFAS score difference between good vs. fair satisfaction was 7.9 (83.9 vs. 78.1) points and that the mean preoperative vs. postoperative change was 30.2 vs. 22.3 points, respectively.

Complication rates after MIS correction vary. A recent systematic review of 1246 patients reported that the overall complication rate of percutaneous HV surgery ranged from 0% to 80%, with a weighted mean of 22.99% [40]. The most common complication was joint stiffness (18.47%), followed by HV recurrence and shortening of the M1 (both 15.2%), material intolerance (10.1%), osteoarthritic changes (9.1%), infection (7.6%), and transfer metatarsalgia (5.4%) [40]. A revision of 317 cases of Inshall–Reverdain distal osteotomy reported 21 cases (6.3%) with different types of complications [8]. Patients who experience a complication are significantly more likely to have a worse MOXFQ index score at 6 months than those who have a normal postoperative recovery [36].

Female patients presenting with HV deformities have a significantly reduced quality of life compared to the general population [1]. Surgical correction of this deformity significantly improves patient quality of life [16]. However, physical and psychological factors of patients may influence the outcomes and recovery from surgery. Even when surgery is performed by an experienced surgeon, a potential remains for patients to experience dissatisfaction and unfavourable outcomes [23].

We used only one functional questionnaire and one PROM questionnaire to assess the patients in this study. However, both MOXFQ and AOFAS have been shown to be highly responsive to clinical changes in the context of HV surgery [32]. These questionnaires are most commonly used to assess clinical results after HV correction [7,14,18,49,50]. Moreover, unlike the AOFAS and MOXFQ, other widely used tools, such as the Short Form 36 questionnaire, have proven to be neither reliable nor responsive enough to detect real changes after forefoot surgery [25].

We did not radiologically assess at any time point possible changes. However, we aimed to study only clinical score changes, and several publications have shown no significant correlation between radiological and clinical findings in patients with HV [16,20,21,36]. Moreover, some authors have reported that predictors of patient satisfaction include subjective outcomes, such as those assessed by the AOFAS score and the Short Form 36 composite quality of life scale, rather than objective radiological outcomes [21].

We only enrolled patients with isolated HV correction, and we do not know whether the results would be different if minor toe corrections were included. However, Lewis et al. [36] reported no significant differences in the scores of the three MOXFQ subscales 2 years after HV correction whether MICA had been performed alone or in association with other procedures. Nevertheless, Miranda et al. [40] pointed out that associated procedures of the lesser toes may affect surgery outcomes, as lateral ray osteotomies may prevent one of the most-feared complications, namely transfer metatarsalgia.

### Limitations

Our study has several limitations. Although large effect sizes were observed in our study, the total number of included patients was relatively small. However, the inclusion criteria restricted the size of the study population. We do not know how the inclusion of patients with complications after surgery may have influenced the results or whether the inclusion of men in the study population may have altered the results. We did not consider demographic/social data (Body mass index, smoking, alcohol habits, etc.) and do not know how these factors might influence our results. We used only one MIS technique; however, there is sufficient recent evidence that the type of operation has no significant influence on the clinical results after HV surgery [15,16,17,18,21]. Moreover, Miranda et al. [40] found in a systematic review that none of the percutaneous techniques were superior. We did not report the follow-up results between the immediate postoperative period and 1 year after the operation, although these data might provide important information. Evidence suggests that most of the clinically important improvements are reached as soon as 3 months after surgery [36]. Over 90% of patients showed an improvement in clinical PROMs 6 months after MICA. Nevertheless, postoperative complications have a negative impact on PROMs 6 months after surgery, although patients can improve over time despite complications [38]. Finally, although our patients did not complain of the operated hallux valgus-related pain, we did not study specific tools such as the visual analogic scale to measure this point.

## 5. Conclusions

We studied a cohort of patients without any complications after MIS for HV correction. Surgeons must inform their patients about possible complications of the operation and how these can influence the outcomes. However, it is also important to inform patients of the expected outcomes when no complications occur. To our knowledge, this is the first study to report this perspective. Although the number of cases needs to be increased and different surgeons and techniques should be included in future studies, this study will help inform patients about the outcomes they can expect over the years if no complications occur. According to our results, the outcomes reported 1 year after MIS correction with the Isham–Reverdin technique in patients without postoperative complications will last without substantial changes for at least 6 years.

## Figures and Tables

**Table 1:** Time points at which the patients were assessed.

Time of Clinical Assessment	Mean (SD)	Min–Max
Preoperative evaluation, days	2.7 (1.1)	1–9
First postoperative evaluation (1.0 years), months	12.6 (1.21)	10–15
Second postoperative evaluation (4.7 years), months	56.8 (5.9)	41–65
Third postoperative evaluation (6.5 years), months	77.1 (8.4)	55–88

Max, maximum; Min, minimum; SD, standard deviation.

**Table 2:** Morphological type of the affected foot of the study population.

Foot Type	Foot Laterality		Total
	Right	Left	
Egyptian	6 (23.1%)	9 (24.3%)	15
Square	10 (38.5%)	13 (35.1%)	23
Greek	10 (38.5%)	15 (40.5%)	25
Total	26	37	63

**Table 3:** Preoperative AOFAS and MOXFQ scores according to the laterality of the affected foot.

Scales	Foot Laterality	Mean (SD)	Min–Max	*p*
MOXFQ_pre	Right (n = 26)	43.23 (10.69)	24–63	0.594
	Left (n = 37)	41.84 (9.76)	23–60	
AOFAS_pre	Right (n = 26)	36.04 (13.34)	10–52	0.480
	Left (n = 37)	34.59 (10.77)	14–52	

AOFAS, American Orthopaedic Foot and Ankle Society; Max, maximum; Min, minimum; MOXFQ; Manchester Oxford Foot Questionnaire; SD, standard deviation.

**Table 4:** Preoperative AOFAS and MOXFQ scores according to the morphological type of the affected foot.

Scales	Foot Type	Mean (SD)	Min–Max	*p*
MOXFQ_pre	Egyptian (n = 15)	42.00 (9.82)	30–56	0.968
	Square (n = 23)	42.83 (6.62)	32–56	
	Greek (n = 25)	42.28 (12.89)	23–63	
AOFAS_pre	Egyptian (n = 15)	35.80 (12.58)	10–52	0.975
	Square (n = 23)	35.13 (11.74)	18–52	
	Greek (n = 25)	34.88 (11.92)	14–50	

AOFAS, American Orthopaedic Foot and Ankle Society; Max, maximum; Min, minimum; MOXFQ; Manchester Oxford Foot Questionnaire; SD, standard deviation.

**Table 5:** Preoperative scores of the W-S, Pain, and S-I subscales according to the laterality of the affected foot.

Items	Foot Laterality	Mean (SD)	Min–Max	*p*
W-S_pre	Right (n = 26)	17.08 (5.20)	7–27	0.739
	Left (n = 37)	16.65 (4.87)	8–27	
Pain_pre	Right (n = 26)	15.88 (3.81)	8–21	0.989
	Left (n = 37)	15.95 (3.67)	8–22	
S-I_pre	Right (n = 26)	10.27 (2.93)	6–16	0.132
	Left (n = 37)	9.24 (2.39)	6–15	

Max, maximum; Min, minimum; SD, standard deviation; S-I, Social Interaction; W-S, Walking–Standing.

**Table 6:** Preoperative scores of the W-S, Pain, and S-I subscales according to the morphological type of the affected foot.

Items	Foot Type	Mean (SD)	Min–Max	*p*
W-S_pre	Egyptian (n = 15)	16.73 (4.30)	9–23	0.997
	Square (n = 23)	16.87 (3.65)	12–27	
	Greek (n = 25)	16.84 (6.39)	7–27	
Pain_pre	Egyptian (n = 15)	15.73 (3.75)	10–20	0.935
	Square (n = 23)	16.39 (2.82)	10–20	
	Greek (n = 25)	15.60 (4.41)	8–22	
S-I_pre	Egyptian (n = 15)	9.53 (2.80)	6–15	0.953
	Square (n = 23)	9.57 (2.13)	7–14	
	Greek (n = 25)	9.84 (3.06)	6–16	

Max, maximum; Min, minimum; SD, standard deviation; S-I, Social Interaction; W-S, Walking–Standing.

**Table 7:** Scores of the MOXFQ and AOFAS scales at the four examined time points.

Scale	Mean (SD)	Min–Max	Scale	Mean (SD)	Min–Max
MOXFQ_pre *^a^	42.41 (10.09)	23–63	AOFAS_pre *^c^	35.19 (11.82)	10–52
MOXFQ_1y *^a,^*^b^	22.95 (10.21)	16–63	AOFAS_1y *^c^	85.03 (12.12)	49–95
MOXFQ_4.7y *^a^	23.35 (10.51)	16–62	AOFAS_4.7y *^c,^*^d^	85.94 (12.67)	45–95
MOXFQ_6.5y *^b^	23.43 (10.42)	16–61	AOFAS_6.5y *^c,^*^d^	84.05 (13.14)	45–95

*^a^ MOXFQ_pre > MOXFQ_i (i = 1y, 4.7y, and 6.5y; *p* < 0.001). *^b^ MOXFQ_1y < MOXFQ_6.5y (*p* = 0.048). *^c^ AOFAS_pre < AOFAS_i (i = 1y, 4.7y, and 6.5y; *p* < 0.001). *^d^ AOFAS_4.7y > AOFAS_6.5y (*p* = 0.002). AOFAS, American Orthopaedic Foot and Ankle Society; Max, maximum; Min, minimum; MOXFQ; Manchester Oxford Foot Questionnaire; SD, standard deviation; y, years.

**Table 8:** Scores of the W-S, Pain, and S-I subscales of the MOXFQ at the four examined time points.

Item	Mean (SD)	Min–Max	Item	Mean (SD)	Min–Max	Item	Mean (SD)	Min–Max
W-S_pre *^a^	16.8 (4.9)	7–27	Pain_pre *^b^	15.9 (3.7)	8–22	S-I_pre *^c^	9.6 (2.6)	6–16
W-S_1y *^a^	10.2 (4.4)	7–27	Pain_1y *^b^	6.9 (3.6)	5–20	S-I_1y *^c^	5.8 (2.6)	4–16
W-S_4.7y *^a^	10.4 (4.5)	7–27	Pain_4.7y *^b^	6.9 (3.5)	5–20	S-I_4.7y *^c^	6.0 (2.8)	4–17
W-S_6.5y *^a^	10.4 (4.5)	7–26	Pain_6.5y *^b^	6.9 (3.4)	5–19	S-I_6.5y *^c^	6.0 (2.7)	4–16

*^a^ W-S_pre > W-S_i (i = 1y, 4.7y, and 6.5y; *p* < 0.001). *^b^ Pain_pre > Pain_i (i = 1y, 4.7y, and 6.5y; *p* < 0.001). *^c^ S-I_pre > S-I_i (i = 1y, 4.7y, and 6.5y; *p* < 0.001). Max, maximum; Min, minimum; MOXFQ; Manchester Oxford Foot Questionnaire; SD, standard deviation; S-I, Social Interaction; W-S, Walking–Standing; y, years.

**Table 9:** AOFAS and MOXFQ scores at preoperative and postoperative (6.5 years) according to the laterality of the affected foot.

**Foot Laterality**	**MOXFQ_Pre**	**MOXFQ_6.5y**	***p***
Right (n = 26)	43.23 (10.69)	23.85 (11.8)	<0.001
Left (n = 37)	41.84 (9.76)	23.14 (9.50)	<0.001
**Foot Laterality**	**AOFAS_Pre**	**AOFAS_6.5y**	***p***
Right (n = 26)	36.04 (13.34)	83.46 (13.28)	<0.001
Left (n = 37)	34.59 (10.77)	84.46 (13.22)	<0.001

AOFAS, American Orthopaedic Foot and Ankle Society; MOXFQ; Manchester Oxford Foot Questionnaire; SD, standard deviation; y, years.

**Table 10:** AOFAS and MOXFQ scores at preoperative and postoperative (6.5 years) according to the morphological type of the affected foot.

**Foot Type**	**MOXFQ_Pre**	**MOXFQ_6.5y**	***p***
Egyptian (n= 15)	42.00 (9.82)	24.33 (12.26)	0.001
Square (n = 23)	42.83 (6.62)	24.87 (10.29)	<0.001
Greek (n = 25)	42.28 (12.89)	21.56 (9.46)	<0.001
**Foot Type**	**AOFAS_Pre**	**AOFAS_6.5y**	***p***
Egyptian (n= 15)	35.80 (12.58)	82.73 (16.11)	0.001
Square (n = 23)	35.13 (11.74)	82.83 (11.51)	<0.001
Greek (n = 25)	34.88 (11.92)	85.96 (12.93)	<0.001

AOFAS, American Orthopaedic Foot and Ankle Society; MOXFQ; Manchester Oxford Foot Questionnaire; SD, standard deviation; y, years.

**Table 11:** Walking–Standing (W-S). Pain and Social Interact (S-I) scores at preoperative and postoperative (6.5 years) according to the laterality of the affected foot.

**Foot Laterality**	**W-S_Pre**	**W-S_6.5y**	***p***
Right (n = 26)	17.08 (5.20)	10.5 (4.97)	<0.001
Left (n = 37)	16.65 (4.87)	10.43 (4.36)	<0.001
**Foot Laterality**	**Pain_Pre**	**Pain_6.5y**	***p***
Right (n = 26)	15.88 (3.81)	7.00 (3.83)	<0.001
Left (n = 37)	15.95 (3.67)	6.86 (3.20)	<0.001
**Foot Laterality**	**S-I_Pre**	**S-I_6.5y**	***p***
Right (n = 26)	10.27 (2.93)	6.35 (3.22)	<0.001
Left (n = 37)	9.24 (2.39)	5.84 (2.34)	<0.001

W-S, Walking–Standing; S-I, Social Interact; pre, preoperative; y, years.

**Table 12:** Walking–Standing (W-S). Pain and Social Interact (S-I) scores at preoperative and postoperative (6.5 years) according to the morphological type of the affected foot.

**Foot Type**	**W-S_Pre**	**W-S_6.5y**	***p***
Egyptian (n = 15)	16.73 (4.30)	10.40 (5.21)	0.003
Square (n = 23)	16.87 (3.65)	11.57 (4.67)	0.001
Greek (n = 25)	16.84 (6.39)	9.48 (4.03)	0.014
Foot Type	**Pain_Pre**	**Pain_6.5y**	***p***
Egyptian (n= 15)	15.73 (3.75)	7.67 (4.19)	<0.001
Square (n = 23)	16.39 (2.82)	7.00 (3.25)	<0.001
Greek (n = 25)	15.60 (4.41)	6.40 (3.18)	<0.001
Foot Type	**S-I_Pre**	**S-I_6.5y**	***p***
Egyptian (n= 15)	9.53 (2.80)	6.27 (3.17)	<0.001
Square (n = 23)	9.57 (2.13)	6.30 (2.69)	<0.001
Greek (n = 25)	9.84 (3.06)	5.68 (2.54)	<0.001

W-S, Walking–Standing; S-I, Social Interact; pre, preoperative; y, years.

## Data Availability

The data that support the findings of this study are available from the corresponding author, G.L.G., upon reasonable request.

## References

[1] Lewis T.L., Ray R., Gordon D.J. (2022). The impact of hallux valgus on function and quality of life in females. Foot Ankle Surg..

[2] Zhu M., Chen J.Y., Yeo N.E.M., Koo K., Rikhraj I.S. (2021). Health-related quality-of-life improvement after hallux valgus corrective surgery. Foot Ankle Surg..

[3] Ray J.J., Friedmann A.J., Hanselman A.E., Vaida J., Dayton P.D., Hatch D.J., Smith B., Santrock R.D. (2019). Hallux valgus. Foot Ankle Orthop..

[4] Biz C., Fosser M., Dalmau-Pastor M., Corradin M., Rodà M.G., Aldegheri R., Ruggieri P. (2016). Functional and radiographic outcomes of hallux valgus correction by mini-invasive surgery with Reverdin-Isham and Akin percutaneous osteotomies: A longitudinal prospective study with a 48-month follow-up. J. Orthop. Surg. Res..

[5] Guo C., Li C., Li X., Xu Y., Cai M., Xu X. (2021). Hallux valgus correction comparing percutaneous oblique osteotomy and open chevron osteotomy at a 2-year follow-up. Orthop. Surg..

[6] Trnka H.-J. (2021). Percutaneous, MIS and open hallux valgus surgery. EFORT Open Rev..

[7] Nair A., Bence M., Saleem J., Yousaf A., Al-Hilfi L., Kunasingam K. (2022). A systematic review of open and minimally invasive surgery for treating recurrent hallux valgus. Surg. J..

[8] Caravelli S., Mosca M., Massimi S., Costa G.G., Lo Presti M., Fuiano M., Grassi A., Zaffagnini S. (2018). Percutaneous treatment of hallux valgus: What’s the evidence? A systematic review. Musculoskelet. Surg..

[9] Castellini J.L.A., Grande Ratti M.F., Gonzalez D.L. (2022). Clinical and radiographic outcomes of percutaneous third-generation double first metatarsal osteotomy combined with closing-wedge proximal phalangeal osteotomy for moderate and severe hallux valgus. Foot Ankle Int..

[10] Isham S.A. (1991). The Reverdin-Isham procedure for the correction of hallux abducto valgus—A distal metatarsal osteotomy procedure. Clin. Podiatr. Med. Surg..

[11] Vernois J., Redfern D.J. (2016). Percutaneous Surgery for Severe Hallux Valgus. Foot Ankle Clin..

[12] Lam P., Lee M., Xing J., Di Nallo M. (2016). Percutaneous Surgery for Mild to Moderate Hallux Valgus. Foot Ankle Clin..

[13] Biz C., Crimì A., Fantoni I., Tagliapietra J., Ruggieri P. (2021). Functional and Radiographic Outcomes of Minimally Invasive Intramedullary Nail Device (MIIND) for Moderate to Severe Hallux Valgus. Foot Ankle Int..

[14] Alimy A.-R., Polzer H., Ocokoljic A., Ray R., Lewis T.L., Rolvien T., Waizy H. (2023). Does minimally invasive surgery provide better clinical or radiographic outcomes than open surgery in the treatment of hallux valgus deformity? A systematic review and meta-analysis. Clin. Orthop. Relat. Res..

[15] Chrea B., Day J., Dean D.M., Cortina R.E., Reilly M., Caolo K.C., Cerrato R.A., Johnson A. (2022). Comparing open vs minimally invasive techniques for the correction of hallux valgus: Clinical and patient reported outcomes. Foot Ankle Orthop..

[16] Hernández-Castillejo L.E., Martínez-Vizcaíno V., Álvarez-Bueno C., Quijada-Rodríguez J.L., Alonso-Galán M., Garrido-Miguel M. (2022). Effectiveness of hallux valgus surgery on improving health-related quality of life: A follow up study. Foot Ankle Surg..

[17] Kaufmann G., Weiskopf D., Liebensteiner M., Ulmer H., Braito M., Endstrasser F., Wagner M., Ban M., Dammerer D. (2021). Midterm results following minimally invasive distal chevron osteotomy: Comparison with the minimally invasive Reverdin-Isham osteotomy by means of meta-analysis. In Vivo.

[18] Patnaik S., Jones N.J., Dojode C., Narang A., Lal M., Iliopoulos E., Chougule S. (2022). Minimally invasive hallux valgus correction: Is it better than open surgery?. Foot.

[19] Xu Y., Guo C., Li X., Xu X. (2022). Radiographic and clinical outcomes of minimally invasive surgery versus open osteotomies for the correction of hallux valgus. Int. Orthop..

[20] Motta L.M., Manchado I., Blanco G., García-Flemate F., González J., Garcés G.L. (2022). Pre- and post-operative relationship between radiological measures and clinical outcomes in women with hallux valgus. J. Clin. Med..

[21] Thiyagarajan H., Lee M., Chen J., Meng N.Y.E. (2022). Predictors of patient satisfaction in hallux valgus surgery. J. Foot Ankle Surg..

[22] Seki H., Suda Y., Takeshima K., Nagashima M., Ishii K. (2022). Patient-reported outcomes of minimally invasive distal linear metatarsal osteotomy for hallux valgus. J. Am. Podiatr. Med. Assoc..

[23] Dismore L.L., van Wersch A., Critchley R., Murty A., Swainston K. (2022). A qualitative study to understand patients’ experiences of their post-operative outcomes following forefoot surgery. Br. J. Pain.

[24] Hernández-Castillejo L.E., Martínez Vizcaíno V., Garrido-Miguel M., Cavero-Redondo I., Pozuelo-Carrascosa D.P., Álvarez-Bueno C. (2020). Effectiveness of hallux valgus surgery on patient quality of life: A systematic review and meta-analysis. Acta Orthop..

[25] Adames D.N.B., González-Lucena G., Ruales J.I.S., Cudos B.G., Ginés-Cespedosa A. (2022). Outcome assessment performance of the SF-36, Manchester-Oxford Foot Questionnaire and AOFAS in forefoot reconstruction surgery. J. Foot Ankle Surg..

[26] De Carvalho K.A.M., Baptista A.D., De Cesar Netto C., Johnson A.H., Dalmau-Pastor M. (2022). Minimally invasive chevron-Akin for correction of moderate and severe hallux valgus deformities: Clinical and radiologic outcomes with a minimum 2-year follow-up. Foot Ankle Int..

[27] Lucattelli G., Catani O., Sergio F., Cipollaro L., Maffulli N. (2020). Preliminary experience with a minimally invasive technique for hallux valgus correction with no fixation. Foot Ankle Int..

[28] Restuccia G., Lippi A., Shytaj S., Sacchetti F., Cosseddu F. (2021). Percutaneous foot surgery without osteosynthesis in hallux valgus and outcomes. Arch. Bone Jt. Surg..

[29] Schrier J.C.M., Palmen L.N., Verheyen C.C.P.M., Jansen J., Koëter S. (2015). Patient-reported outcome measures in hallux valgus surgery. A review of literature. Foot Ankle Surg..

[30] Sawah A., Zemenova S., Haque R., Ridley D., Abboud R.J., Wang W., Harrold F. (2021). Forecasting posttreatment outcome of hallux valgus surgery patients. Foot Ankle Int..

[31] Morley D., Jenkinson C., Doll H., Lavis G., Sharp R., Cooke P., Dawson J. (2013). The Manchester-Oxford Foot Questionnaire (MOXFQ): Development and validation of a summary index score. Bone Jt. Res..

[32] Dawson J., Boller I., Doll H., Lavis G., Sharp R., Cooke P., Jenkinson C. (2011). The MOXFQ patient-reported questionnaire: Assessment of data quality, reliability and validity in relation to foot and ankle surgery. Foot.

[33] Holme T.J., Sivaloganathan S.S., Patel B., Kunasingam K. (2020). Third-generation minimally invasive chevron Akin osteotomy for hallux valgus. Foot Ankle Int..

[34] McMurrich W., Peters A., Ellis M., Shalaby H., Baer G., MacDonald D., McKinley J.C. (2020). MIS Distal Metatarsal Metaphyseal Osteotomy in the treatment of metatarsalgia: MOXFQ patient reported outcomes. Foot.

[35] Lewis T.L., Ray R., Miller G., Gordon D.J. (2021). Third-generation minimally invasive chevron and Akin osteotomies (MICA) in hallux valgus surgery: Two-year follow-up of 292 cases. J. Bone Jt. Surg. Am..

[36] Lewis T.L., Ray R., Gordon D.J. (2022). Minimally invasive surgery for severe hallux valgus in 106 feet. Foot Ankle Surg..

[37] Lewis T.L., Robinson P.W., Ray R., Dearden P.M.C., Goff T.A.J., Watt C., Lam P. (2023). Five-year follow-up of third-generation percutaneous chevron and Akin osteotomies (PECA) for hallux valgus. Foot Ankle Int..

[38] Lewis T.L., Ray R., Gordon D.J. (2022). Time to maximum clinical improvement following minimally invasive chevron and Akin osteotomies (MICA) in hallux valgus surgery. Foot Ankle Surg..

[39] Klein E.E., Wirt C., Greenley R., Weil L.S., Weil L., Fleischer A.E. (2022). Do patient personality traits and self-reported physical and psychosocial symptoms help to predict hallux valgus surgery outcomes?. J. Foot Ankle Surg..

[40] Miranda M.A.M., Martins C., Cortegana I.M., Campos G., Pérez M.F.M., Oliva X.M. (2021). Complications on percutaneous hallux valgus surgery: A systematic review. J. Foot Ankle Surg..

[41] Baumhauer J.F., Nawoczenski D.A., DiGiovanni B.F., Wilding G.E. (2006). Reliability and validity of the American Orthopaedic Foot and Ankle Society clinical rating scale: A pilot study for the hallux and lesser toes. Foot Ankle Int..

[42] Kitaoka H.B., Alexander I.J., Adelaar R.S., Nunley J.A., Myerson M.S., Sanders M. (1994). Clinical rating systems for the ankle-hindfoot, midfoot, hallux, and lesser toes. Foot Ankle Int..

[43] Dawson J., Coffey J., Doll H., Lavis G., Cooke P., Herron M., Jenkinson C. (2006). A patient-based questionnaire to assess outcomes of foot surgery: Validation in the context of surgery for hallux valgus. Qual. Life Res..

[44] Del Vecchio J.J., Ghioldi M.E., Chemes L.N., Dealbera E.D., Brue J., Dalmau-Pastor M. (2021). Percutaneous, intra-articular, chevron osteotomy (PeICO) for the treatment of mild-to-moderate hallux valgus: A case series. Int. Orthop..

[45] Lai M.C., Rikhraj I.S., Woo Y.L., Yeo W., Ng Y.C.S., Koo K. (2018). Clinical and radiological outcomes comparing percutaneous chevron-Akin osteotomies vs open scarf-Akin osteotomies for hallux valgus. Foot Ankle Int..

[46] Kaufmann G., Dammerer D., Heyenbrock F., Braito M., Moertlbauer L., Liebensteiner M. (2019). Minimally invasive versus open chevron osteotomy for hallux valgus correction: A randomized controlled trial. Int. Orthop..

[47] Nilsdotter A.-K., Cöster M.E., Bremander A., Cöster M.C. (2019). Patient-reported outcome after hallux valgus surgery—A two year follow up. Foot Ankle Surg..

[48] Chan H.Y., Chen J.Y., Zainul-Abidin S., Ying H., Koo K., Rikhraj I.S. (2017). Minimal clinically important differences for American Orthopaedic Foot & Ankle Society score in hallux valgus surgery. Foot Ankle Int..

[49] Bia A., Guerra-Pinto F., Pereira B.S., Corte-Real N., Oliva X.M. (2018). Percutaneous osteotomies in hallux valgus: A systematic review. J. Foot Ankle Surg..

[50] Malagelada F., Sahirad C., Dalmau-Pastor M., Vega J., Bhumbra R., Manzanares-Céspedes M.C., Laffenêtre O. (2019). Minimally invasive surgery for hallux valgus: A systematic review of current surgical techniques. Int. Orthop..

